# Screening for risky behaviour and mental health in young people: the YouthCHAT programme

**DOI:** 10.1186/s40985-017-0068-1

**Published:** 2017-10-13

**Authors:** Felicity Goodyear-Smith, Rhiannon Martel, Margot Darragh, Jim Warren, Hiran Thabrew, Terryann C. Clark

**Affiliations:** 1 Department of General Practice and Primary Health Care, Faculty of Medical and Health Science, University of Auckland, PB 92019, Auckland, New Zealand; 2 Department of Computer Science, Faculty of Science, University of Auckland, PB 92019, Auckland, New Zealand; 3 Department of Psychological Medicine, Faculty of Medical and Health Science, University of Auckland, PB 92019, Auckland, New Zealand; 4 School of Nursing, Faculty of Medical and Health Science, University of Auckland, PB 92019, Auckland, New Zealand

**Keywords:** Screening, Mental health, Adolescent, Depression, Anxiety, Self-injurious behaviour, Risk reduction behaviour, Suicide, eHealth

## Abstract

**Background:**

The prevalence of mental health concerns and risky health behaviours among young people is of global concern. A large proportion of young people in New Zealand (NZ) are affected by depression, suicidal ideation and other mental health concerns, but the majority do not access help. For NZ indigenous Māori, the burden of morbidity and mortality associated with mental health is considerably higher. Targeted screening for risky behaviours and mental health concerns among youth in primary care settings can lead to early detection and intervention for emerging or current mental health and psychosocial issues. Opportunistic screening for youth in primary care settings is not routinely undertaken due to competing time demands, lack of context-specific screening tools and insufficient knowledge about suitable interventions. Strategies are required to improve screening that are acceptable and appropriate for the primary care environment. This article outlines the development, utilisation and ongoing evaluation and implementation strategies for YouthCHAT.

**YouthCHAT:**

YouthCHAT is a rapid, electronic, self-report screening tool that assesses risky health-related behaviours and mental health concerns, with a ‘help question’ that enables youth to prioritise areas they want help with. The young person can complete YouthCHAT in the waiting room prior to consultation, and after completion, the clinician can immediately access a summary report which includes algorithms for stepped-care interventions using a strength-based approach. A project to scale up the implementation is about to commence, using a co-design participatory research approach to assess acceptability and feasibility with successive roll-out to clinics. In addition, a counter-balanced randomised trial of YouthCHAT versus clinician-administered assessment is underway at a NZ high school.

**Conclusion:**

Opportunistic screening for mental health concerns and other risky health behaviours during adolescence can yield significant health gains and prevent unnecessary morbidity and mortality. The systematic approaches to screening and provision of algorithms for stepped-care intervention will assist in delivering time efficient, early, more comprehensive interventions for youth with mental health concerns and other health compromising behaviours. The early detection of concerns and facilitation to evidence-based interventions has the potential to lead to improved health outcomes, particularly for under-served indigenous populations.

## Background

### Mental health problems and risky behaviours are common in New Zealand’s young people

Mental health concerns and health risk behaviours, such as tobacco, alcohol and other drug use, physical inactivity and sexual risk behaviours, are initiated and often consolidated during adolescence. Depressive and anxiety disorders and self-harm (including suicide) are three of the top five causes of loss of disability-adjusted life years in 15–19 years old [1]. A quarter of New Zealand’s (NZ’s) young people are affected by depression and anxiety, and over half engage in hazardous drinking by the age of 18 [2, 3]. Young people with long-term physical conditions are also at increased risk of mental health problems, particularly anxiety and depression [4–6]. Suicide is the leading cause of death for NZ youth aged 15–24 years and second leading cause for those aged 10–14 years [7]. For Māori, NZ’s indigenous population, the burden of morbidity and mortality associated with mental health is considerably higher, with Māori males living in deprived areas having the highest rates of suicide [8, 9] and disproportionately high rates of depressive symptoms [8, 10].

### Early detection and treatment of these problems is important for both individuals and society

Family and friends play a critical part in helping young people through difficult periods in their lives; however, the development of mental health issues are frequently only recognised when a crisis occurs [11, 12]. Early identification of emerging mental health or psychosocial issues gives health professionals the opportunity to work with the young person to recognise and nurture their own positive qualities, assets and pro-social relationships. Evidence shows that developing problem-solving skills and fostering help-seeking behaviours can make young people more resilient during difficult times [13].

The World Health Organization recognises the need for appropriately targeted services to address the unique health and social needs of youth [1, 14]. Policy documents from numerous NZ organisations have underscored the importance of delivering more integrated youth services, with collaboration between social, health, education and other sectors to address the challenges, and intervening earlier when problems emerge, and advocate for reduction in inequity among Māori and other vulnerable youth [11, 15–21].

Despite the availability of effective treatments, 75% of NZ’s adolescent population do not access help from primary care to address these concerns [2, 22]. Early detection and intervention is paramount for youth who have developed, or who are at risk of developing, mental health conditions; however, this cannot occur unless those who are experiencing these issues are identified and offered help [23–26]. Assessment and early intervention for youth mental health and psychosocial issues in primary care must use user-friendly screening tools that can be adapted to be usable and sustainable in different clinical contexts [27, 28].

The most common access to primary health care for NZ youth is through general practice [23, 29]. For Māori and others living in areas of high deprivation, health care delivered through school-based and youth-specific health clinics can improve access significantly [30–33]. Youth with mental health issues are often seen in primary health care in terms of problem or risk needing management [34, 35]; however, they do have strengths and abilities enabling them to be involved in the development of their plan of care [34, 35], which suggests that preventative screening and the provision of self-management tools may be a beneficial approach. Young people can be helped to develop healthy coping strategies through nurturing their resilience, developed from positive characteristics in their lives such as family and peer support, connectedness to their community and culture, and involvement in groups where they feel accepted and valued [36–38]. A strength-based approach helps young people to develop support systems and coping strategies to facilitate better lifestyle choices and promote positive adaptation when faced with future challenges [36, 39, 40].

Depressive disorders place a high financial strain on NZ’s economy and are the major contributing factor in youth suicide and youth mental health issues. Risk-taking behaviours developed during adolescence contribute to long-term poor health and socio-economic problems [25, 34, 41]. Untreated mental health and behavioural issues can have personal costs on youth, their families and local communities; there are also enormous societal costs associated with the flow on effects of untreated disorders [42, 43]. A NZ longitudinal study that followed children with mental illness and their siblings without mental illness for a period of 40 years found that as adults, those with mental illness had more time off work for sick leave, earned 20% less, and had fewer assets [44]. They were also 11% less likely to be married. The research suggests that there is a lifetime cost of NZ$300,000 of family income and a total lifetime economic cost for all affected of 2.1 trillion dollars (based on the assumption that one in 20 adults experiences mental health problems in childhood). NZ research has found that psychiatric disorder among young adults is associated with lower income and living standards and reduced workforce participation [45].

### Screening may aid early detection of mental health problems and risky behaviour in young people

Health professionals in general practice, school and youth clinics are well placed to undertake opportunistic screening of young people for mental health and psychosocial issues and to provide early intervention. Despite the burden of mental health morbidity in the community, there is evidence that health professionals in general practice do not opportunistically discuss emotional or behavioural issues with youth [46, 47], unless the issues are serious or actively raised by the young person [48]. Health professionals in general practice give reasons for this lack of engagement as being shortage of time, experience and skills in youth mental health, and inadequate knowledge of suitable interventions once such concerns have been identified [2, 49].

Young people attending *primary care* commonly have more than one psychosocial or mental health issue that needs attention [27, 34, 50, 51]. Identifying such issues relies on a thorough psychosocial assessment by the clinician, during which the young person must disclose personal information to someone whom they may only have recently met [24]. While there are several screening tools available [27], they can be time-consuming and may not all be suitable for different settings [33, 52].

The HEEADSSS assessment (Home, Education, Eating, Activities, Drugs and Alcohol, Sexuality, Suicide/Depression, Safety) is a clinician-administered interview-based assessment of youth that can help identify mental health and substance use problems [53]. Currently, all NZ year 9 (13–14 years old) students in low decile (areas of high social deprivation) schools are expected to be assessed for well-being via HEEADSSS. While HEEADSSS offers a straightforward, holistic and gradual approach to assessing young people across many domains [53], it is a face-to-face assessment, not a screening tool. Its drawbacks include its lack of validation, cost of resourcing, lack of integration with young person’s primary care provider and time required for administration, which can be in excess of 40 min and may take up to 2 h [52].

Screening and case-finding are terms sometimes used synonymously [54], both involving the early detection of a condition. However, screening and case-finding may differ with respect to their setting and the expectations of their populations [55]. Screening generally refers to testing an asymptomatic population for the presence of a condition which if identified can lead to early intervention reducing subsequent morbidity or mortality. Case-finding involves seeking early detection of a condition when a patient attends for an unrelated concurrent disorder, and may or may not be symptomatic. For a specific condition, testing will depend on a number of criteria including the age and gender of the patient and the presence of any risk factors which might increase their likelihood of being a positive case (increase the pre-test probability).

For screening to be justified, the WHO and the Journal of the American Medical Association evidence-based medicine working group require that it is an important health problem, with a suitable acceptable test and a clear diagnosis, that the benefits outweigh any harms and that early intervention is effective and cost-effective [56, 57]. The US Preventive Services Task Force similarly directs that there must be an accurate test for the condition and scientific evidence that screening can prevent adverse outcomes.

In general, there is good evidence for targeted screening in primary care settings for risk behaviours such as tobacco use [58], alcohol [59] and illicit drug use [60], problem gambling [61] and physical inactivity [62], and mental health issues including depression [63] and anxiety [64], given appropriate intervention is then available. Young people are more vulnerable to developing risk behaviours and mental health issues which can be carried on into adulthood. For health outcomes to be improved, early detection of emerging or current issues and appropriate intervention is paramount [2, 24–26], and thus targeted screening for risk behaviours and mental health issues among youth attending primary care is justified.

While studies using accepted screening criteria may have been conducted on the effectiveness of screening, there still may not be consensus on whether or not to screen. Even meta-analyses with the same research question, such as the evidence for screening for depression, can result in opposing recommendations [65]. Screening criteria act as guidelines, but different components may be given different weightings. Ultimately, the decision to systematically screen or case-find or not will be directed by value judgements and the importance placed on various aspects, including consideration of the specific population in question and availability of potential interventions. Thus, the implementation of a national mental health screening tool at a local level may not succeed if community and cultural priorities regarding health and well-being are not understood [66]. For implementation of interventions to be successful, there must be consultation and input from the local community, so that their health needs can be met [66–68].

### Electronic screening may have a role to play in detecting mental health problems and risky behaviour in young people

Electronic screening (e-screening) has been shown to provide consistent results, lead to more disclosure and reduce staff time [69, 70]. There is emerging research that suggests youth prefer to complete self-assessment via electronic means [24, 71–73]. E-screening is associated with youth disclosing sensitive information without fear of being judged, structuring their thoughts and prioritising the issues for which they wanted help. Young people feel more in control and have more input into their ongoing care [24], making it more likely that they will see any intervention as beneficial [35].

### YouthCHAT is a potentially useful screening instrument for identifying mental health problems and risky behaviour in young people

YouthCHAT is a youth-specific, self-administered, holistic risk behaviour and mental health e-screening and intervention planning programme that has been developed in NZ. The aim of this article is to discuss its rationale, development, progressive implementation and potential impact on youth health and well-being.

## YouthCHAT

### Description of YouthCHAT

YouthCHAT is a composite screener for psychosocial issues that was developed from an adult-oriented screening tool, the electronic Case-Finding and Help Assessment Tool (eCHAT) in NZ. eCHAT is a self-report rapid (5–15 min) tool screening for substance misuse, problem gambling, depression, anxiety, exposure to abuse, difficulty controlling anger and physical inactivity in primary care settings [74, 75]. A key feature is the help question, which enables patients to indicate areas where they would like help, gauge their readiness to change, and prioritise issues where they have problems in more than one domain [76–78]. Initially developed, evaluated and validated as a paper tool [79–83], the electronic version enables branching logic. Positive responses for smoking, alcohol and other drug use lead directly to the WHO Alcohol, Smoking and Substance Involvement Screening Test (ASSIST) [84], for depression to the Patient Health Questionnaire - 9 (PHQ-9) [85] and for anxiety to the GAD-7 [86]. With the electronic format, the results are able to be communicated immediately to a relevant care provider and include a summary of the assessments and help question responses as a preface to the detailed responses. There is also the potential for development of electronic decision support and stepped-care algorithms.

Young people self-administer YouthCHAT electronically prior to their consultation. Once completed, the health provider/clinician is immediately able to access a summary report indicating which modules screened positive, the severity (e.g. from depression PHQ-A score) and whether help is sought. Review of this summary facilitates a conversation between the young person and health provider (for example family physician or nurse) and the shared decision-making of an action plan. YouthCHAT provides a guide to effective evidence-based interventions using a stepped-care management model ranging from self-help (for example helpline numbers, handouts and web addresses for psychoeducation and e-therapies), to GP or primary care nurse brief interventions or provision of relevant medication (such as nicotine replacement), to referral to community agencies, and services, and finally referral to secondary care (mental health and drug and alcohol services). This approach engages youth and empowers them to have input into their management plans, encourages them to develop strengths and interests and increases the chances of effective intervention.

### Development of YouthCHAT

The first version of YouthCHAT was developed in 2015. Additional modules relating to sexual health (concerns about sexual orientation/identity, risky sexual behaviours and unwanted sexual activity) were added to the existing nine modules (smoking, drinking and other drug use, gambling, depression, anxiety, exposure to abuse, anger control and physical inactivity). The ASSIST for drinking and recreational drug use was replaced by the youth-friendly Substances and Choices Scale (SACS), developed and validated in NZ [87] and the PHQ-9 with PHQ-A (modified for adolescents). It was also made available in both English and Māori languages. It was successfully implemented in rural clinic for rural youth, especially Māori, and favourably received by both young patients and clinic staff [88].

In 2016, YouthCHAT was updated with stakeholder assistance from a low decile high school to match the modules of the face-to-face HEEADSSS assessment. This involved adding three modules on eating and conduct disorders and areas of stress in their lives.

Development of both eCHAT and YouthCHAT has involved stakeholder engagement including patients, clinical staff, community agencies and Māori in a number of different forums [79, 81, 83, 89, 90].

### Current clinical utilisation and research of YouthCHAT

Implementation of YouthCHAT is soon to be underway for use in NZ settings with large Māori populations in nurse-led youth clinics, school-based clinics, and general practice. It is anticipated that a successful roll-out will be associated with improved health and social outcomes through early identification and intervention of mental health concerns, improvement in youth resilience and help-seeking behaviour and an acceptable and time-saving and cost-effective strategy for clinicians to screen for mental health concerns and ultimately improve equity for young Māori [23, 26, 91]. A framework has been developed for scaling up the implementation of YouthCHAT e-screening into primary care environments across other primary healthcare settings.

The feasibility and acceptability of the programme is being researched using an implementation and co-design participatory research approach with a mixed method design [92]. While randomised controlled trials provide evidence about the use of a specific intervention in a controlled setting with a very specific group of patients, this evidence may not be fully transferrable to complex interventions for use in actual clinical contexts [93, 94].

An implementation approach allows the research team to work with the young people and clinic staff to identify aspects that limit or encourage the use of YouthCHAT in each specific clinical context [95]. Strategies to overcome obstacles to its implementation across different settings can be developed and evaluated in order to develop a successful formula to scale up the use of YouthCHAT across a range of primary care contexts. And data from interviews and focus groups, rates of detection for each domain of YouthCHAT, health-seeking behaviour and provision of brief interventions or referrals to secondary care mental health services can be assessed before and after YouthCHAT implementation.

**Fig. 1:**
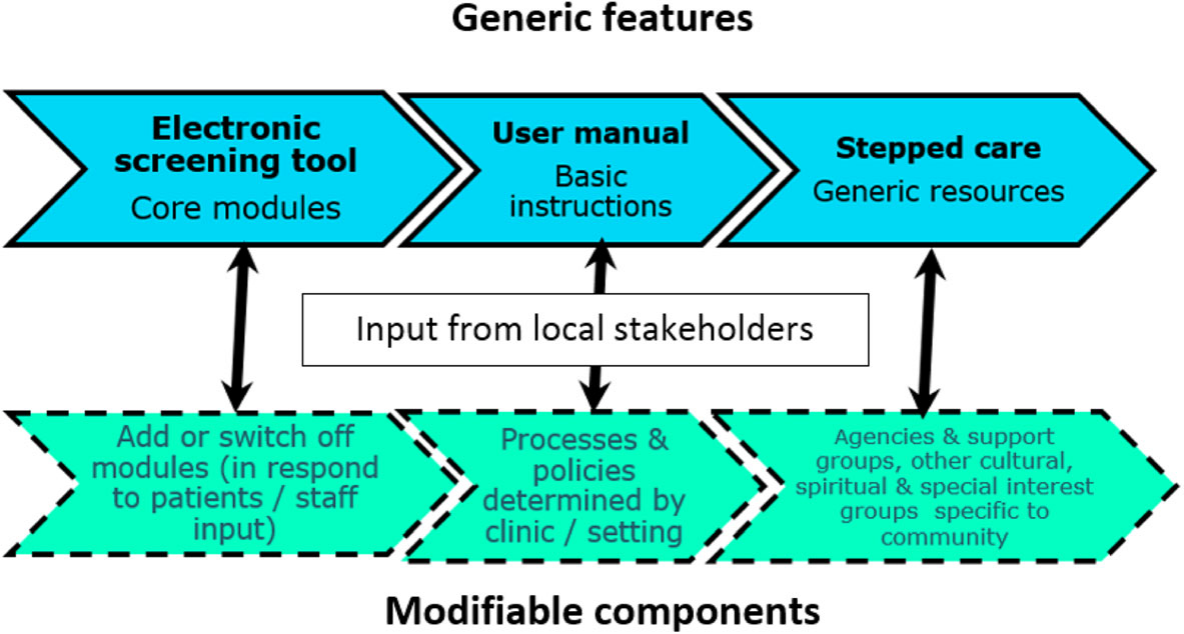
Implementation framework

A co-design participatory research approach [96] helps ensure that end-user feedback supports the development of a sustainable implementation of YouthCHAT. This process involves consultation and partnership between researchers, clinicians, young people, support staff, managers and policymakers in research planning and adaptation of the programme in response to feedback. The tailoring of YouthCHAT to each specific setting involves consultation with clinical staff and key community members and cultural leaders. Modifiable elements include specific screening modules, determining the screening processes and criteria for that clinic, and identifying local resources, agencies, cultural and community supports that might be included in the stepped-care intention package. Input from the community enables modification of the programme in response to relevant socioeconomic and contextual factors of the targeted region (see Fig. 1). Furthermore, their engagement with, and shared ownership of, the programme optimises the chance of successful implementation.

YouthCHAT has the potential to overcome barriers associated with opportunistic mental health screening of youth, including HEEADSSS assessment, while providing a similar holistic assessment of mental health and lifestyle issues. A counter-balanced randomised trial of YouthCHAT versus HEEADSSS is currently underway at a NZ high school [97].

YouthCHAT is also the screening tool being used in a NZ National Science Challenge project ‘Health Advances through Behavioural Intervention Technologies’ (HABITS) which is developing Internet and app-based psychological interventions to be used by young people, either independently or in association with their youth health worker [98]. The project hopes to increase detection of problems among young people and promote new ways for them to access evidence-based interventions for common mental health concerns. By increasing access to therapy available ‘anywhere and anytime’, HABITS aims to improve mental health in the short-term and demonstrate improved long-term outcomes with better school retention and employment and reduced substance abuse, antisocial behaviour and mental health problems.

### Potential impact of YouthCHAT

Early detection of mental health issues and risky health behaviours in young people may lead to many downstream social as well as financial benefits, including improved physical health, reduction in school dropout, increased employment, less suicides, more successful relationships including marriage, and a reduction in crime rates.

The average cost of keeping a New Zealander in prison for 12 months in 2011 was $91,000 or $250 per day [99]. Many incarcerated individuals have substance abuse issues and face problems such as anger management, gambling, mental health problems and more. The longer these problems are left untreated, the more entrenched and harder to treat they become. By focusing on identification and early intervention of high-risk youth, it is possible that YouthCHAT can mitigate these mental health and behaviour issues that are so often the basis for offending and incarceration. The cost ramifications of this can be significant. For example, by helping just one youth avoid a 5-year prison sentence by overcoming mental health and behaviour issues that lead to incarceration, YouthCHAT could save NZ $455,000.

A national generic framework for mental health services can provide a base from which context-specific processes and policies can be developed [68]. Sharing components of youth mental health in primary care common to all areas can save time and money at the local level. Considerations such as costs and benefits, workforce development and reaching vulnerable groups may help inform creation of services. A national framework can also help develop consistent standards of care based on best practice and measuring of outcomes.

However, the implementation of national mental health strategies at the local level may not succeed if community and cultural priorities regarding health and well-being are not understood [66]. For implementation of interventions to succeed, there must be consultation and input from the local community, so that their health needs can be met [66–68].

With sufficient coverage, YouthCHAT data could also be used for monitoring the prevalence of mental health concerns among youth within a population—this would help to prioritise services and workforce development in areas of high need. Collated YouthCHAT data can provide information on identification of mental health and addiction issues in youth, and inform provision of appropriate stepped-care interventions, from self-management to practice-delivered brief interventions and medications, community-level services and secondary care services. Collated anonymised data can be provided at various levels from individual practices to practice networks and regional health services. This can assist in appropriate provision of mental health and addiction services to align service provision with population need, and improve services through benchmarking. A data analytics portal has been designed and prototyped to support a range of users including practice managers, clinical directors and health policy analysts based on initial YouthCHAT field trial data [100].

## Conclusions

Opportunistic screening for mental health concerns and other risky health behaviours during adolescence can yield significant health gains and prevent unnecessary morbidity and mortality, including self-harm and suicide. The systematic approaches to screening and provision of algorithms for stepped-care intervention will assist in delivering time efficient, early, more comprehensive interventions for youth with mental health concerns and other health compromising behaviours. The early detection of concerns and facilitation to evidence-based interventions has the potential to lead to improved health outcomes, particularly for under-served indigenous populations.

## Abbreviations

ASSIST: Alcohol, Smoking and Substance Involvement Screening Test
eCHAT: Electronic Case-finding and Help Assessment Tool
GAD-7: General Anxiety Disorder - 7
HABITS: Health Advances through Behavioural Intervention Technologies
HEEADSSS: Home, Education, Eating, Activities, Drugs and Alcohol, Sexuality, Suicide/Depression, Safety
NZ: New Zealand
PHQ-9: Patient Health Questionnaire - 9
PHQ-A: PHQ-9 modified for Adolescents
SACS: Substances and Choices Scale
WHO: World Health Organization
